# ddRAD sequencing: an emerging technology added to the biosecurity toolbox for tracing the origin of brown marmorated stink bug, *Halyomorpha halys* (Hemiptera: Pentatomidae)

**DOI:** 10.1186/s12864-021-07678-z

**Published:** 2021-05-17

**Authors:** Juncong Yan, Gábor Vétek, Chandan Pal, Jinping Zhang, Rania Gmati, Qing-Hai Fan, Disna N. Gunawardana, Allan Burne, Diane Anderson, Rebijith Kayattukandy Balan, Sherly George, Péter Farkas, Dongmei Li

**Affiliations:** 1 Plant Health and Environment Laboratory, Ministry for Primary Industries, PO Box 2095, Auckland, 1140 New Zealand; 2 Institute of Plant Protection, Department of Entomology, Hungarian University of Agriculture and Life Sciences, Villányi út 29-43, Budapest, H-1118 Hungary; 3 MARA-CABI Joint Laboratory for Bio-safety, Institute of Plant Protection, Chinese Academy of Agricultural Sciences, No. 2 Yuanmingyuan West Road, Beijing, 100193 P.R. China; 4 Biosecurity Science and Risk Assessment, Ministry for Primary Industries, Wellington, New Zealand; 5 Plant Health and Environment Laboratory, Ministry for Primary Industries, P.O. Box 14018, Christchurch, 8544 New Zealand

**Keywords:** BMSB, SNP, Population genetics, Invasion, Biosecurity, ddRADSeq, Restriction digestion

## Abstract

**Background:**

Brown marmorated stink bug (BMSB), *Halyomorpha halys* (Hemiptera: Pentatomidae) is native to East Asia but has invaded many countries in the world. BMSB is a polyphagous insect pest and causes significant economic losses to agriculture worldwide. Knowledge on the genetic diversity among BMSB populations is scarce but is essential to understand the patterns of colonization and invasion history of local populations. Efforts have been made to assess the genetic diversity of BMSB using partial mitochondrial DNA sequences but genetic divergence on mitochondria is not high enough to precisely accurately identify and distinguish various BMSB populations. Therefore, in this study, we applied a ddRAD (double digest restriction-site associated DNA) sequencing approach to ascertain the genetic diversity of BMSB populations collected from 12 countries (2 native and 10 invaded) across four continents with the ultimate aim to trace the origin of BMSBs intercepted during border inspections and post-border surveillance.

**Result:**

A total of 1775 high confidence single nucleotide polymorphisms (SNPs) were identified from ddRAD sequencing data collected from 389 adult BMSB individuals. Principal component analysis (PCA) of the identified SNPs indicated the existence of two main distinct genetic clusters representing individuals sampled from regions where BMSB is native to, China and Japan, respectively, and one broad cluster comprised individuals sampled from countries which have been invaded by BMSB. The population genetic structure analysis further discriminated the genetic diversity among the BMSB populations at a higher resolution and distinguished them into five potential genetic clusters.

**Conclusion:**

The study revealed hidden genetic diversity among the studied BMSB populations across the continents. The BMSB populations from Japan were genetically distant from the other studied populations. Similarly, the BMSB populations from China were also genetically differentiated from the Japanese and other populations. Further genetic structure analysis revealed the presence of at least three genetic clusters of BMSB in the invaded countries, possibly originating via multiple invasions. Furthermore, this study has produced novel set of SNP markers to enhance the knowledge of genetic diversity among BMSB populations and demonstrates the potential to trace the origin of BMSB individuals for future invasion events.

**Supplementary Information:**

The online version contains supplementary material available at 10.1186/s12864-021-07678-z.

## Background

The brown marmorated stink bug (BMSB), *Halyomorpha halys* (Stål, 1855) (Hemiptera: Pentatomidae) is a highly polyphagous pest with a wide host range [1]. It can cause severe damage to agricultural crops worldwide [2, 3], and in 2010 alone was responsible for a loss of more than 37 million USD in agricultural products in North America [4]. The native range of BMSB is China (including Taiwan), Japan, and the Korean peninsula [5–7]. To date, BMSB has been reported from more than 30 countries [8], including almost all states in the USA [2, 4], multiple countries in Europe [9–16] and Chile [17]. Climate modelling studies indicates its potential range could expand further, including South and Central America, Southern Africa, Southern Australia, and the North Island of New Zealand [9, 18].

In the past decades, BMSB has invaded and established in a range of countries irrespective of the environmental conditions [2, 4, 10–17, 19]. Adaptive evolutionary changes and/or ecological adaptation in a new region has made this pest a successful global invader and its recent invasion history can shed light on that. However, in-depth genetic information of BMSB at the population level is scarce. Such information on the genetic diversity of BMSB can enhance our understanding of their population structure and global invasion history. This could also assist in constructing a global genetic population structure of BMSB and develop a potential strategy to trace the country of origin for BMSB individuals intercepted at the border or in post-border scenarios in biosecurity settings.

BMSB is a serious pest for agriculture and horticulture and can be a social nuisance. As agricultural exports play a significant role in New Zealand’s Gross Domestic Product, the establishment of the pest would be highly detrimental to the country. BMSB has increasingly been intercepted at the New Zealand border. Since is first intercepted in 2005 [20], the frequency of interceptions have been increasing due to the rise of international travelling and trade [21]. There have been 2009 recorded interceptions of BMSB since 2005 at the New Zealand border (up to November 2020) [20]. Therefore, it is important to study the genetic structure and composition of BMSB populations to assist in tracing its origin and predicting the potential invasive pathways.

To date, nearly all published studies for tracing the origin of BMSB utilized PCR based molecular methods and focused on small regions on mitochondrial DNA (mtDNA), such as the COI (Cytochrome c oxidase I) and/or COII (Cytochrome c oxidase II) genes [16, 19, 22–24]. mtDNA is highly variable between species and can potentially provide sufficient resolution to identify genetic differences between species [25]. Since mtDNA is inherited maternally and lacks recombination, the resolution of mitochondria-derived genetic divergence is generally not sufficient to differentiate between individuals in a population [26]. Therefore, there is the need to study the genome-wide, high-resolution markers among BMSB populations from their native and invaded regions. The study will be able to discern genetically distinct populations thus allowing us to trace the geographical origin of BMSBs within an interception scenario. This calls for an innovative method to explore the genetic diversity within BMSB populations on a genome-wide scale.

The detection of different genetic markers is crucial for studying genetic diversity. Recently, a high-throughput sequencing-based method (HTS) has replaced traditional gel-based experiment to discover genetic markers [27]. RADseq (Restriction-site Associated DNA Sequencing), is often applied for genome-wide SNP (Single Nucleotide Polymorphism) identification in large genomes because of its relatively low cost and high-throughput [28]. The RADseq technique utilises one (or more) restriction enzyme(s) to digest the whole genome into short genomic fragments that are then subjected to high-throughput DNA sequencing [28]. Restriction site-associated DNA markers provide a well-established basis for population genetics, as they are sensitive to both SNPs and insertion or deletion events (indels) in genomes [29]. So far, RADseq has been widely used in population genetic studies for many taxa including plants [30], and animals [28]. Double digest Restriction-site Associated DNA (ddRAD) sequencing uses two restriction enzymes to allow greater control of the genomic regions sampled for sequencing and more reproducible recovery of sequenced regions [31]. Therefore, in this study, we applied ddRAD sequencing (ddRADseq) to explore the genetic diversity among BMSB specimens collected from 41 populations across 12 countries.

## Results

### EcoR I-Msp I restriction enzyme pair was suitable for ddRAD sequencing

To select the most suitable restriction enzyme (RE) pairs for digesting BMSB genomes, in silico test using 15 combinations of REs against the BMSB genome scaffolds were conducted. The simulation revealed that more than 100 K fragments produced from most of the RE pairs selected except the pairs, MseI-MluCI, MspI-PstI and EcoRI-PstI (Table 1). Since the genome used for the test was consisted of scaffolds instead of a complete genome, the simulation results might not reflect the real situation. A pilot ddRADseq in vitro experiment was conducted with genomic DNA samples derived from two BMSB individuals (one male and one female). Of the 15 pairs of REs used for the in silico test, nine different pairs of REs were selected for ddRADseq. After the HiSeq run, approximately 2 Gb of raw RADseq sequencing data were generated for each individual. The EcoRI-MspI restriction enzyme pair recovered the highest number of genetic variances (i.e. high quality SNPs) after highly stringent SNP quality control (QC) filtering, thus was selected it as the most suitable pair of restriction enzymes for digesting the BMSB genome via ddRAD sequencing (Table 1, Additional file 1).

**Table 1:** Summary of the in silico and in vitro tests of RE pairs for ddRADseq

RE pairs	In silico: numbers of Segment^a^	In vitro: numbers of SNPs^b^
MspI-NlaIII	556,040	969
EcoRI-NlaIII	508,739	311
PetI-NlaIII	481,590	Null
MseI-PstI	303,697	17
EcoRI-MseI	296,739	20,136
EcoRI-MluCI	267,087	Null
MspI-MseI	265,748	27,871
PstI-MluCI	259,942	Null
MspI-MluCI	222,963	16,369
MseI-NlaIII	182,338	3909
NlaIII-MluCI	148,911	2135
EcoRI-MspI	118,867	28,328
MseI-MluCI	86,829	Null
MspI-PstI	86,012	Null
EcoRI-PstI	31,417	Null

Note: ^a^Prediction of the DNA segments of 300–500 bp against the BMSB genome scaffold

^b^Two replicates were used for each RE pairs. The number showed the shared SNPs between two replicates. Null indicates not tested

### ddRAD sequencing statistics and SNPs estimation

In total, 399 ddRAD sequencing datasets were obtained from the BMSB individuals, which yielded a total of 3.6 billion raw paired end reads (2 × 150 bp) (min: 4 million, max: 40 million and median: 7.6 million paired end reads per sample). On average, 9 million raw paired end reads were generated for each individual. The 3′ end adaptors of raw reads were trimmed and low quality reads were discarded. Using quality-trimming of the sequence data, 387,629 SNPs were estimated from 399 BMSB individuals. A highly stringent QC criterion was applied for filtering the SNPs, and only those loci that were shared by all the individuals were retained. This resulted with 1775 high confidence biallelic SNPs from 389 individuals. Further analysis showed that the 1775 SNPs were distributed in 484 scaffolds and 1–20 SNPs were detected in each of those scaffolds with average 3.7 SNPs per scaffold (Additional file 2). The 1775 SNPs were used for the subsequent analysis of genomic diversity and population structure.

### Genetic clusters were observed among the BMSB populations

**Fig. 1:**
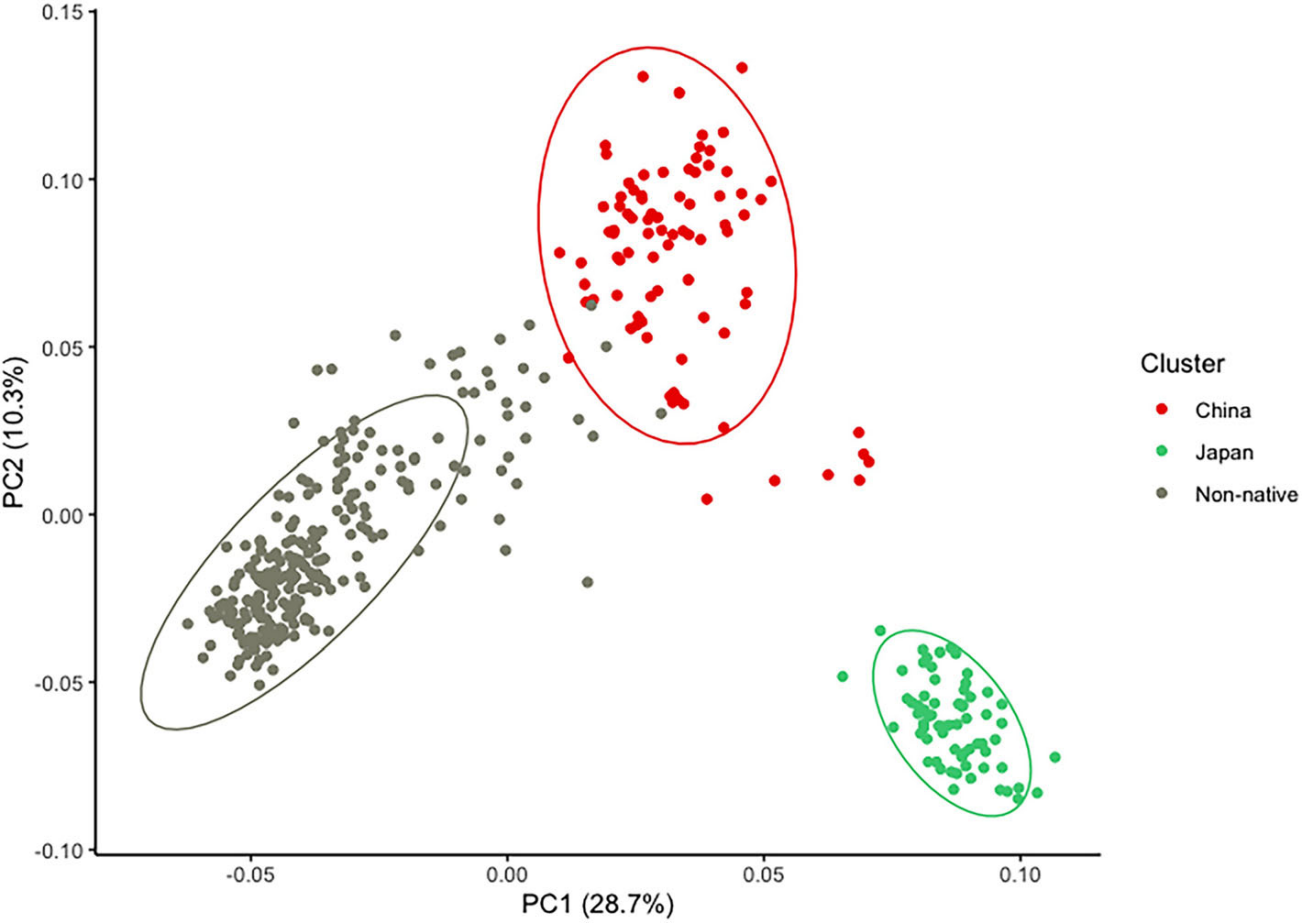
Principal component analysis (PCA) plot using 1775 SNPs of 389 individuals. Each point represents the SNP profile of an individual. The colour represents the country where the individuals were collected from. X axis represents the variance explained by PC2 (10.3%), and Y axis represents the variance explained by PC1 (28.7%). The figure was created using R package ggplot [32]

At least three genetic clusters comprising China, Japan, and the invaded countries (Austria, Chile, Georgia, Hungary, Italy, Romania, Serbia, Slovenia, Turkey, and the USA) were revealed by Principal Component Analysis (PCA) using the SNP data generated from 389 BMSB individuals (Fig. 1). All BMSB individuals from Japan formed an isolated cluster, whereas BMSBs collected from the invaded countries were genetically closer to those of China. Analysis using 484 representative SNPs (one from each scaffold) produced similar result (Additional file 3).

### Individuals from the same geographical region were genetically linked

**Fig. 2:**
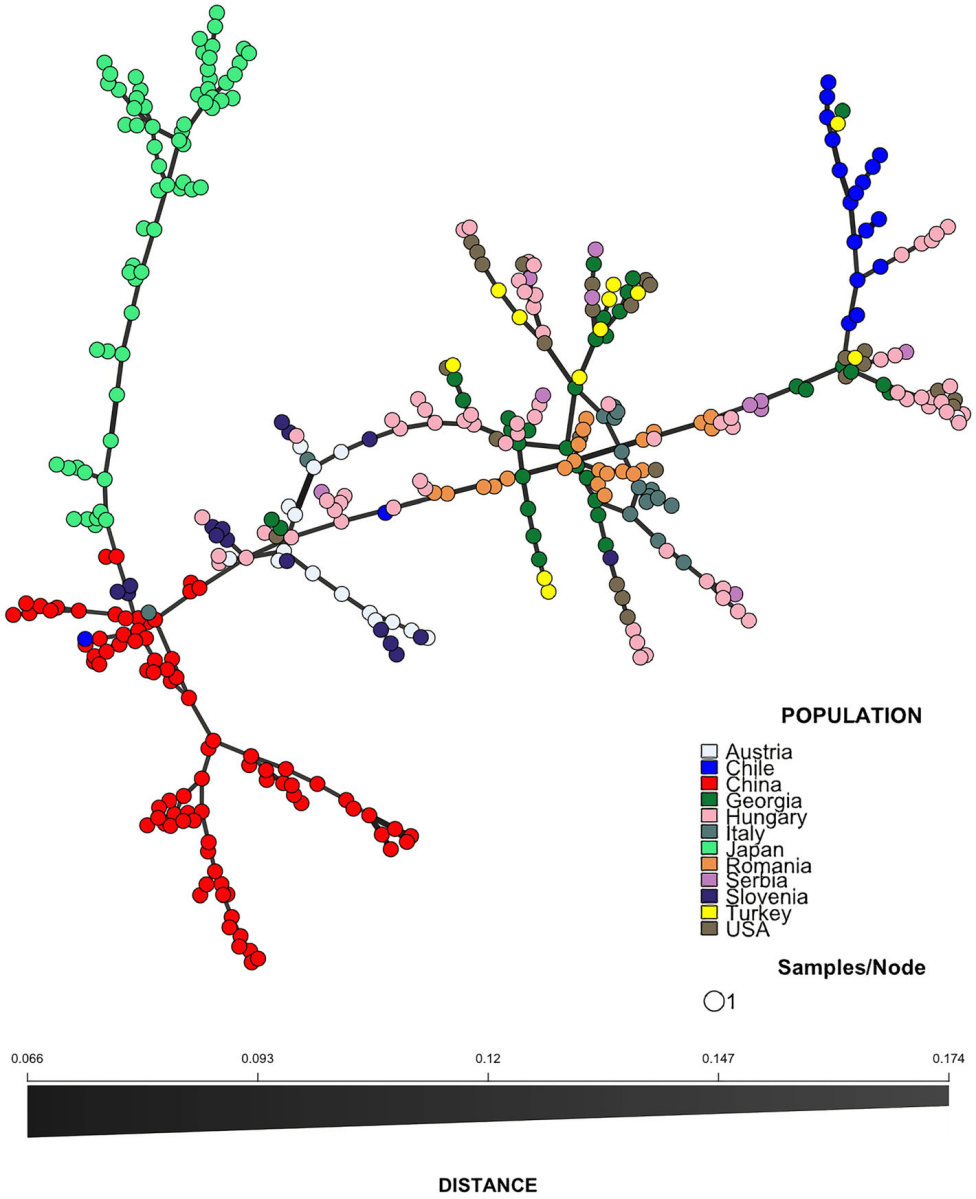
Minimum spanning networks (MSN) of BMSB individuals. The analysis was based on 1775 SNPs derived from 389 individuals of 12 geographical groups comprising Austria, Chile, China, Georgia, Hungary, Italy, Japan, Romania, Serbia, Slovenia, Turkey, and the USA. Each node represents an individual specimen and the edge indicates the genetic distance (dissimilarity: fast pairwise distances) between the individuals. The colour in each circle represents the countries where the samples were collected from. The figure was created using R package poppr [33]

**Fig. 3:**
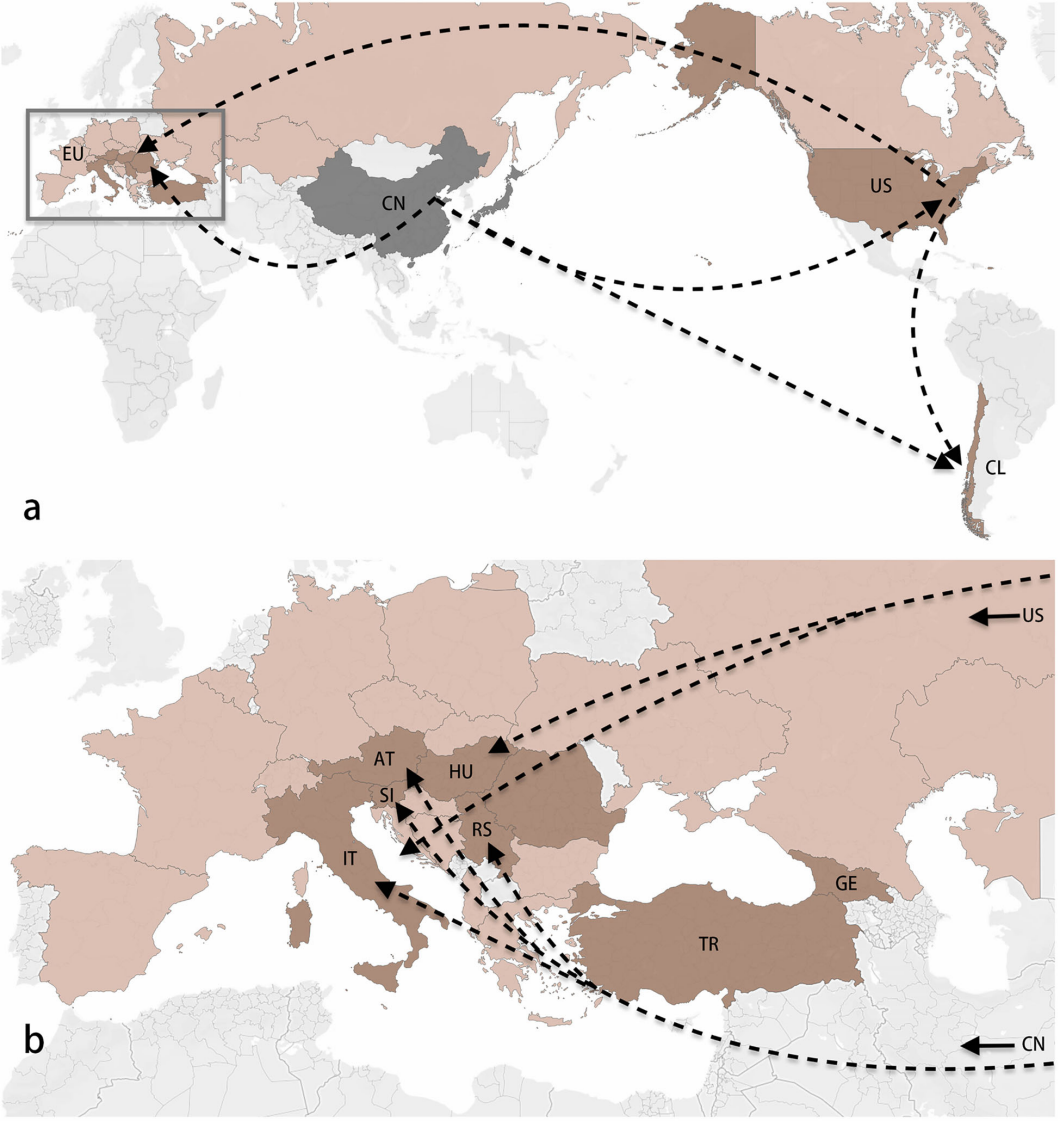
A roadmap of the most likely BMSB invasion pathways constructed based on the results of this study. The dark grey and brown/light orange colour on the map represent the native countries of BMSB and the invaded countries, respectively. Those countries where BMSB were included in this study are showing in dark grey and brown. The arrows with the dotted lines indicate the possible pathway of invasion. Countries were labelled with the country ISO code (https://countrycode.org/). AT: Austria; CL: Chile; CN: China; GE: Georgia; HU: Hungary; IT: Italy; JP: Japan; SI: Slovenia; RS: Serbia; TR: Turkey and US: United States. Figure 3a showed the overall BMSB invasive pathways while the Fig. 3b is the enlarged map for the European countries. The figure was created using Tableau based on the results from the SNPs data

To further emphasise the outcome of genetic clustering pattern via principal component analysis, minimum spanning networks (MSN) were constructed using the SNPs profile of each individual, and genetic variability was visualised among the population lineages (Fig. 2). The MSN showed that all the individuals from China were genetically linked together in the network, which also applies to the individuals from Japan (Fig. 2). There was a genetic divergence among the BMSB individuals from native regions of China and Japan, while those of invaded countries were more closely related in the network. One individual from Chile was found in the same clade of the Chinese samples, suggesting that this BMSB specimen might have originated from a recent invasion from China. The rest of the Chilean samples were distantly related from those in China and Japan but were more closely related to the samples from the European/USA groups, indicating that those possibly originated from secondary invasions from European/USA regions (Figs. 2 and 3). The MSN also showed that one individual from Italy and three from Slovenia were genetically linked to the Chinese populations, whereas the rest from these two countries were more closely related to those from European and the USA, suggesting multiple invasions might have occurred (Figs. 2 and 3).

### Genetic distance between native populations of China and Japan was relatively higher

**Fig. 4:**
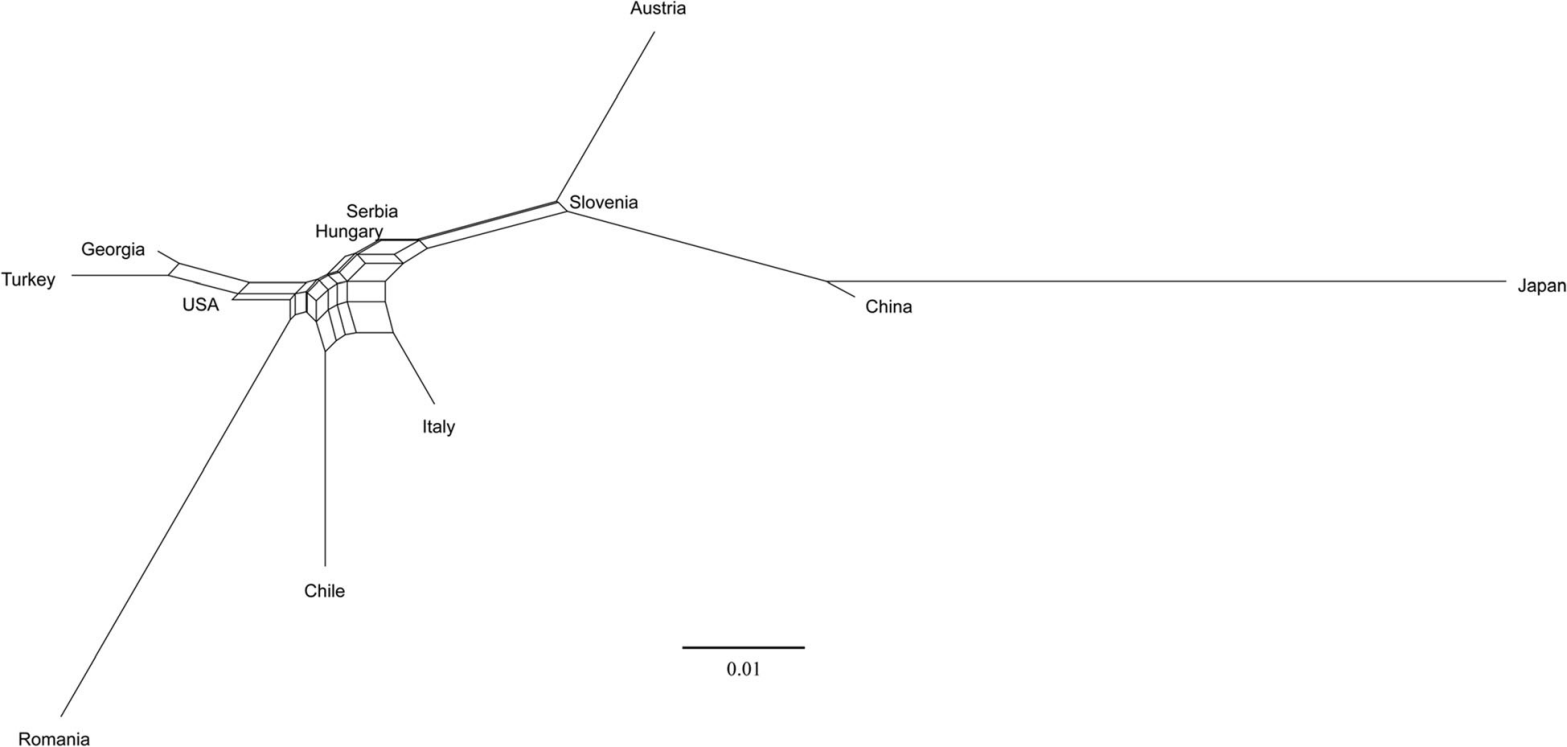
The Neighbour-net tree of 12 geographical groups. The phylogenetic tree was constructed using SplitsTree 4 [34] based on genetic distances of population pairwise F_ST_ values. The tree shows the evolutionary history of each BMSB population

Population genetic divergence in the form of pairwise F_ST_ revealed significant (*p* < 0.05) genetic differences (except for that between China and Serbia) among 12 geographical groups or countries, with F_ST_ value ranging from 0.0006 between BMSB populations from Hungary and Serbia, to 0.2084 between BMSB populations from Japan and Romania (Table 2). We also observed that the genetic distance between native populations of China and Japan was moderately higher (F_ST_ = 0.0847) than that between the populations of China and many other BMSB-invaded countries, such as Slovenia (F_ST_ = 0.0379). Similarly, the genetic distance between the invaded populations in the USA and Chile was relatively low (F_ST_ = 0.0393) compared to the genetic distance between BMSB populations in Chile and the native regions, China (F_ST_ = 0.0984) and Japan (F_ST_ = 0.1765). Moreover, the F_ST_ value between the BMSB populations from the neighbouring countries was very small, for example, Turkey and Georgia (F_ST_ = 0.0165); Austria and Slovenia (F_ST_ = 0.0203); Hungary and Serbia (F_ST_ = 0.0006) (Table 2). A Neighbour-net tree constructed using the F_ST_ pairwise values among the individuals from the 12 countries revealed the similar relationships among the BMSB populations from the 12 countries (Fig. 4). The tree depicted the overall relationships of the populations and showed that Chinese and Japanese populations were clustered together, but genetically different. The populations from the invaded countries were genetically linked, but the populations from Romina formed a long branch, indicating the genetic separation from those of the other countries studied (Fig. 4). It also demonstrated that the BMSB from the adjoining countries, i.e. Turkey/Georgia, Austria/Slovenia, Hungary/ Serbia, are more closely related with each other and are likely from the same origin (Fig. 4).

**Table 2:** The group pairwise F_ST_ (Fixation index) between the BMSB populations from 12 countries

	JP	CN	HU	RS	US	CL	SI	IT	RO	TR	AT
CN	0.0847										
HU	0.1437	0.0639									
RS	0.1338	0.0476*	0.0006								
US	0.1626	0.0843	0.0130	0.01226							
CL	0.1765	0.0984	0.0394	0.0405	0.0393						
SI	0.1027	0.0379	0.0249	0.01052	0.0420	0.0671					
IT	0.1517	0.0786	0.0263	0.02892	0.0394	0.0441	0.0376				
RO	0.2084	0.1333	0.0609	0.06616	0.0593	0.0913	0.0912	0.0773			
TR	0.1747	0.0970	0.0332	0.02977	0.0164	0.0567	0.0640	0.0574	0.0937		
AT	0.1398	0.0601	0.0440	0.03359	0.0630	0.0944	0.0203	0.0657	0.1182	0.0918	
GE	0.1682	0.0901	0.0241	0.02107	0.0147	0.0595	0.0516	0.0419	0.0737	0.0165	0.0778

Note: Asterisk (*) indicates no statistically significant difference (*p* > 0.05). Fst value ranges from 0 to 1, where 0 means no genetic difference (i.e. similar) and 1 means high difference (isolated populations). Values close to zero indicate the populations are sharing their genetic structure and has minimal difference between them. Countries were labelled with the country ISO code (https://countrycode.org/). AT: Austria; CL: Chile; CN: China; GE: Georgia; HU: Hungary; IT: Italy; JP: Japan; SI: Slovenia; RS: Serbia; TR: Turkey and US: United States

### Five genetic clusters exist in the BMSB populations

**Fig. 5:**
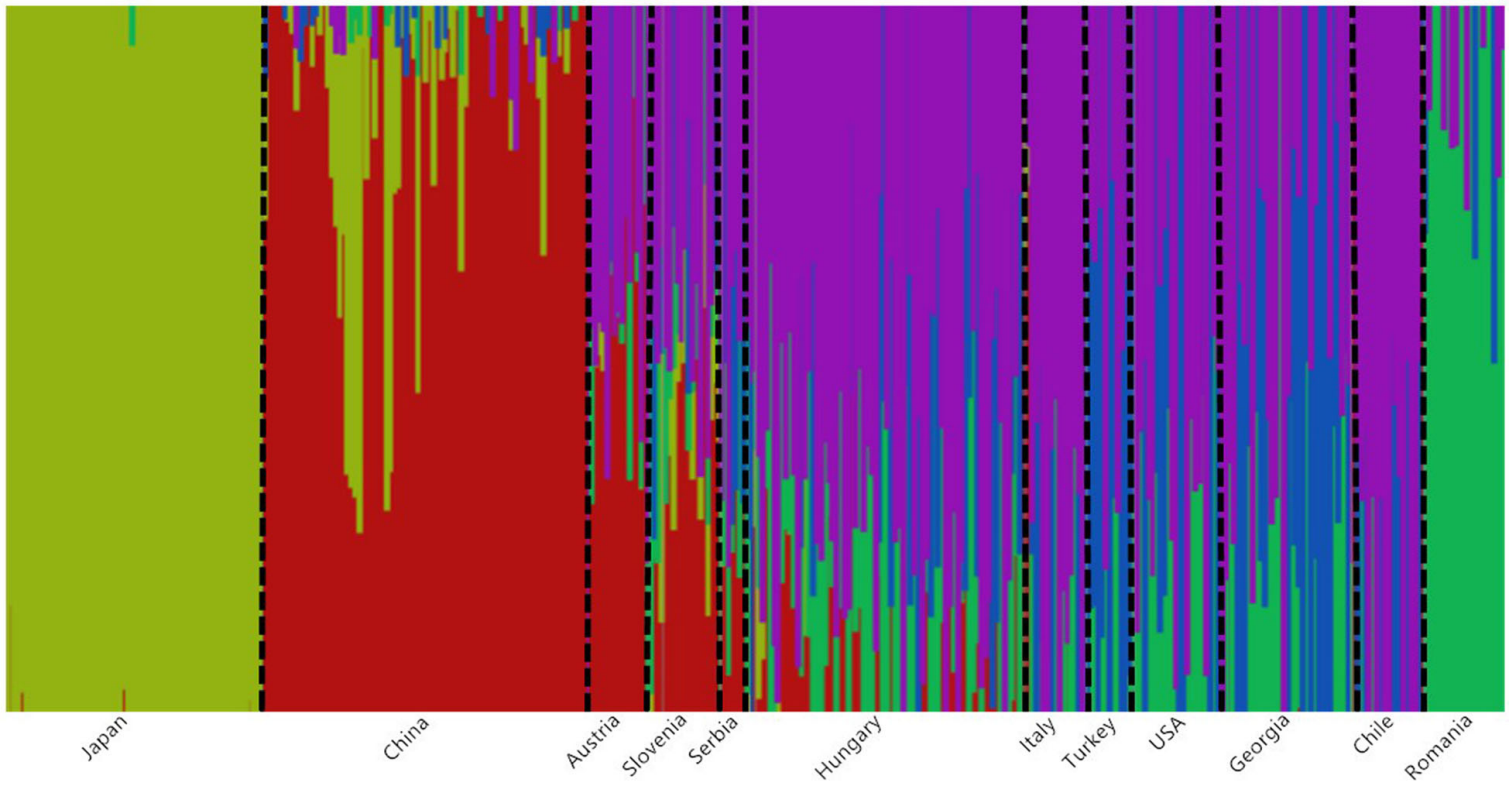
The fastSTRUCTURE bar plot showing the genetic clusters. The assignment probabilities (K = 5; the number of populations or clusters that the samples are best divided into) of each genotyped individual of BMSB from 12 different geographical regions were shown. Each pixel column on x-axis is one individual sample. The proportion of genetic components belonging to different genetic clusters for each individual is represented on the y-axis. Different colours in a bar represent proportion of the genomic component in a sample derived from different populations. Dotted line was used to divide BMSB populations from different countries

Furthermore, insights into the BMSB genetic diversity were unravelled by population genetic structure analysis using fastSTRUCTURE. This analysis expanded the results of PCA (Fig. 1) and provided more in-depth clustering for the BMSB populations from the invaded countries. This analysis predicted the presence of at least three genetic clusters within the BMSB-invaded countries (Fig. 5). The first cluster comprises of populations from the USA, Italy, Chile, Turkey, Georgia, and Hungary (Cluster 1), the second one is formed by Romania (Cluster 2), and the third one is formed by Austria, Serbia and Slovenia (Cluster 3). The BMSB populations from China (Cluster 4) and Japan (Cluster 5) were clearly separated from the invasive populations (Fig. 5).

Fst analysis of the five genetic clusters (Table 3) showed that the genetic distance was moderately higher (Fst > 0.05) among China, Japan, Cluster 2 and Cluster 1, and was lower (Fst < 0.05) between China and Cluster 3 (Table 3). The AMOVA (Analysis of molecular variance) of genetic distance for the samples from the 12 countries allowed a partitioning of three levels (Table 4). The proportion of variation attributable to within country differences was 90.35% while they were only 7.09 and 2.56% occurred among clusters and among countries within the clusters, respectively. The genetic differences among and within the cluster and countries were significant (*p* < 0·05). Therefore, the results indicate that the individuals from one country are more genetically different within them than that the difference they have with the other countries.

**Table 3:** The pairwise F_ST_ (Fixation index) among the five genetic clusters

	Cluster 1	Cluster 2	Cluster 3	Cluster 4
**Cluster 2**	0.0556			
**Cluster 3**	0.0241	0.0845		
**Cluster 4**	0.0666	0.1333	0.0421	
**Cluster 5**	0.1402	0.2084	0.1153	0.0847

Note: All the values are statistically significant difference (*p* > 0.05). Fst value ranges from 0 to 1, where 0 means no genetic difference (i.e. similar) and 1 means high difference (isolated populations). Values close to zero indicate the populations are sharing their genetic structure and has minimal difference between them. The five clusters are: Cluster 1: Chile, Georgia, Hungary Turkey, Italy and the USA; Cluster 2: Romania; Cluster3: Austria, Serbia and Slovenia; Cluster 4: China and Cluster 5: Japan

**Table 4:** Analysis of molecular variance (AMOVA) the five genetic clusters derived from the 389 individuals from the 12 countries

Source of variation	d.f.	SS	Variance components	% variation
Among clusters	4	11,128.09	15.079	7.09
Among countries within clusters	7	3177.27	5.436	2.56
Within countries	784	150,556.74	192.0366	90.35

The calculations were based on SNPs from ddRADseq data. Note: d.f. (Degrees of freedom): the number of independent values on which the estimate is based on. SS (sum of squares): the sum of squared differences of each observation from the mean. The results showed that most of the variation in the dataset was found among the individuals within countries. The five clusters are: Cluster 1: Chile, Georgia, Hungary Turkey, Italy and the USA; Cluster 2: Romania; Cluster3: Austria, Serbia and Slovenia; Cluster 4: China and Cluster 5: Japan

The heterozygosity analysis was showed that the Observed Heterozygosity (H_o_) and the Expected Heterozygosity (H_e_) for all the countries are not very high, around 0.2 (Additional file 7). The H_o_ of Japan is smaller than the H_e_, suggesting that the populations in this country is under inbreeding (isolation). Conversely the H_o_ is bigger than the H_e_ in the other countries, indicating that an isolate-breaking effect is happening, and interbreeding is occurring among those populations.

## Discussion

To the best of our knowledge, this is the most comprehensive population genomic study so far to unravel the genetic diversity and population structure of BMSB. The study utilised ddRAD sequencing to enhance the knowledge of global BMSB genetic diversity and invasion history. We identified a suitable restriction enzyme pair for genomic digestion of BMSB genome for ddRAD sequencing study, which will be useful in future applications. The ddRAD data were analysed using a combination of approaches, including principal component analysis (PCA), phylogenetic analysis and population structure analysis to elucidate the population structure and genetic diversity among the BMSB populations. The present study unambiguously proved that the BMSB populations in the two native regions of China and Japan were genetically distinct. Many BMSB populations from the invaded countries were genetically closer to those of China. Conversely, the Japanese BMSB populations were isolated and showed genetically less similar to those from the invaded countries. Overall, this study has provided a remarkable resolution in unravelling the population structure and estimating the genetic relatedness among the BMSB populations from a set of native and invaded regions of the world.

### Genetics isolation between the BMSB populations from China and Japan

The PCA and MSN analyses showed that the Japanese BMSB populations were isolated from the other populations studied here. The fastSTRUCTURE analysis showed that population structures of the BMSB samples from China and Japan were derived predominantly from different genetic components (Fig. 5). These results suggested that there is genetic isolation for the BMSB populations of the two countries. Xu et al. 2014 [22] also observed genetic divergence between the BMSB populations from China and Japan via haplotype analysis of mitochondrial COII and 12S/CR gene regions. One possible explanation for such phenomenon could be the geographical distance between the isolated populations. The width of Tsushima Strait is from 41.6 km to 222 km which is the distance between mainland China and Japan [35], and the average non-stop flight distance of BMSB is less than 1 km [36]. Therefore, active spread of BMSB individuals from China to Japan is unlikely. We cannot rule out the possibility of the BMSB hitchhiker capability, however this study suggest that there is limited migration among BMSB populations between the two countries. It has been shown that northern Japan or western Korea were potentially the origin of BMSB based on climate matching models [23], but Xu et al. 2014 disagreed with this conclusion and their results implied north China as the ancestral clades. They also demonstrated that China and Japan populations were predicted as one population during the glacier stage [22]. Therefore, future studies by sampling more BMSB samples from wider regions will be required to draw a reliable conclusion.

### Estimation of the BMSB invasion pathway

BMSB populations from the invaded countries are more closely related to China than that of Japan. For example, the USA populations with Fst of 0.084 to the Chinese populations and 0.16 to the Japanese populations, indicating that the USA populations originated more likely from China (Fig. 3), but Japan. The results are consistent with the findings of a previous study by Valentin et al. 2017 [37]. Moreover, there were large genetic differences (0.15 < F_ST_ < 0.25) between the BMSB populations in Japan and the invaded countries of Chile, Georgia, Italy, Romania, Turkey, and the USA (Table 2). Therefore, we can conclude that the BMSB populations in the six invaded countries might not have originated from Japanese populations. The smallest F_ST_ values of 0.0006 between the BMSB populations from Hungary and Serbia, the neighbouring countries, imply they were possibly from the same origin. The Fst analysis also indicated that the BMSB populations from Turkey and Georgia (Table 2) were more likely shared the same source and there were gene flow among them. It is also possible for the BMSB populations from Austria and Slovenia (Table 2).

Moreover, the PCA (Fig. 1, Additional files 3 and 4) indicated that the established populations in Chile, Hungary, Georgia, Italy, Turkey and the USA belonged to one genetic cluster. The population structure analysis using fastSTRUCTURE supported this conclusion that those populations had similar genetic components (Fig. 5). The neutrality test (Fu’s Fs) in our recent study [38] for these populations was negative (*p* < 0.05), indicating that these established populations have been undergoing population expansion stage [39].

One individual BMSB sample from the Italy cohort was genetically closely related to the Chinese population (Fig. 2), suggesting the possible recent arrival of that BMSB from China (Figs. 2 and 3). The rest of the BMSB samples from Italy were more closely related to the USA and other European populations, suggesting that multiple BMSB invasions have occurred in Italy in the past [16, 40]. Such phenomenon was also observed in Chile [17]. Most of the BMSB specimens from Chile, except one individual, were genetically close to each other (Figs. 2 and 3), implying that there were at least two separate invasions of BMSB in Chile.

### Predication of the BMSB genetic cluster

The population structure analysis of BMSB indicated that there were at least five genetic clusters identified among the BMSB populations studied here. Besides the genetic clusters of native BMSB populations from China and Japan, there were three genetic clusters in the BMSB-invaded countires. The analysis showed that the BMSB populations from the USA, Georgia, Turkey, Italy, Hungary, and Chile were more likely belong to one genetic cluster due to their similar genetic structure. Since first detected in the USA in 1996 [41], BMSB has invaded many neighbouring countries. In Europe, BMSB was first recorded in Switzerland in 2004 [14, 21, 42], then spread to many countries such as Germany, France, Italy, Georgia and Hungary [43–45]. Therefore, based on the timeline of reports, BMSB invasion might have started in the USA (1996) [2], then Europe (2004) and Chile (2017) [17], though a more transparent history of invasion remains unknown and needs further investigation.

Another interesting phenomenon is that the genetic structures between BMSB populations originating from Slovenia, Austria, and Serbia and the Chinese populations were more genetically related than those between the Chinese and the other European populations (Fig. 5). This suggests that BMSB found in these three European countries could have recently been invaded directly from China (Figs. 3 and 5) rather than from one of their neighbouring countries in Europe. In saying that, the population genetic structure analysis also showed that the BMSB populations from Slovenia, Austria, and Serbia contained the components of the Europe-established BMSB populations (Fig. 5). This analysis indicated that there was inbreeding between the BMSB individuals from the adjoined established European BMSB populations and the Chinese populations (Figs. 3 and 5). This result indicated that these BMSB populations were still under early stage of invasion, which supports the findings of haplotype analysis using COI and COII of BMSB samples from the same populations in our previous work [38].

BMSB was first reported in Romania in 2015 [10] and have spread around the country [46]. The F_ST_ values between the BMSB samples from Romania and the two native countries, China, and Japan, were 0.1333 and 0.2084, respectively, which were larger than the F_ST_ values from other invaded populations. The population structure analysis showed that Romanian BMSB samples had a unique genetic structure, which indicates that this group could have been invaded from a place of origin that was not sampled in this study. Therefore, this study further emphasised the importance of sampling. Overall, this study covered a wide range of areas including wide BMSB sampling from the native regions and the invaded countries, although we still missed samples from some countries, such as Korea, Greece, Canada and Switzerland. Therefore, further study of ddRADseq of BMSB samples from those countries will provide more insights into genetic diversity of the BMSB populations and facilitate in inferring a more in-depth invasion pathway.

### Novel genetic analysis for the BMSB populations from the invade countries

This study also provided novel genetic information on the BMSB populations for some of the European countries, such as Austria, Serbia, Slovenia, and Georgia. Since BMSB was detected in Austria [47] and Serbia [48] in 2015 and Slovenia [49] in 2017 and Turkey [50] in 2019, to the best of our knowledge, no genetic studies have been reported on those populations, and this study shows the first effort to characterise the BMSB populations for these countries. The results showed that the BMSB populations from Turkey were more likely the next generation of the BMSB from the USA whereas those populations from Austria, Serbia and Slovenia were the hybrids of Chinese and USA populations (Fig. 5). The results obtained here will be useful for future monitoring of the pest and assessing the potential pathways of invasion in those countries.

In comparison with the mitochondrial based (i.e. COI and COII) haplotypes analyses, here we show that ddRADseq can provide a higher resolution in identifying and characterising different BMSB genetic populations. As an alternative, analysis of genome-wide SNPs via whole genome sequencing can be applied for deeper understanding the BMSB genome and genetic diversity. However, it is still more expensive due to the large size of the BMSB genome (~ 1 Gbp) [51]. Therefore, cost effective approach such as the RAD-based approach with restriction enzymes (ddRAD) used in this study can provide a higher resolution assessment of genomic diversity and population structure among BMSB populations. Taken together, we recommend applying genome-wide SNP markers-based study using ddRAD to explore the genomic diversity in insects and other eukaryotic organisms in future applications. Most importantly, in biosecurity settings, our study will help to trace the geographic origin of the BMSB samples, irrespective of their life stages, and sex, that are intercepted at border. The advancement and findings resulted from our study will not only help in claiming the BMSB country freedom status to New Zealand but also will greatly assist in the decision making during an incursion and response events.

## Conclusions

This study demonstrates that the restriction enzymes pair of EcoRI and MspI is suitable for generating restriction-associated DNA fragments for identifying SNPs within the BMSB genome through ddRAD sequencing. Via population genomic analyses using ddRADseq data, we detected remarkable genetic diversity among different BMSB populations studied in both the native and invaded countries. We believe that this study could also assist in studying the invasion history of BMSB populations and tracing the potential geographical origin of unknown BMSB intercepted at the border or in post-border scenarios in biosecurity settings using the population structure model. Thus, ddRAD sequencing going to be an invaluable emerging technology added into the biosecurity toolbox for tracing the geographic origin of insects at the border/post-border in the near future.

## Methods

### BMSB specimen collection and DNA extraction

BMSB samples were collected from 41 regions in 12 countries, namely Austria, Chile, China, Georgia, Hungary, Italy, Japan, Romania, Serbia, Slovenia, Turkey, and the USA (Table 5, Additional file 5). The field-collected samples were preserved in plastic vials containing 95% ethanol and stored at − 20 °C until DNA extraction could be performed. Total genomic DNA was extracted from each individual using QIAGEN DNeasy® Blood & Tissue Kit with QIAGEN RNase A treatment (Qiagen, Valencia, CA, USA). Briefly, head and thorax or abdomen was ground using sterile plastic pestle in 1.5 mL tube with ATL buffer with Proteinase K and RNase A added, and then incubated at 56 °C overnight. The DNA extraction followed the manufacturer’s instructions and the DNA was eluted in 150 μl AE buffer. DNA quality was assessed using NanoDrop™ spectrophotometer (ThermoFisher Scientific, USA) and quantified using QuantiFluor™ system (ThermoFisher Scientific, USA). It was followed by an assessment for DNA shearing on a 1% agarose gel against 1 Kb Plus DNA ladder (Invitrogen™, CA, USA) in TAE buffer stained with SYBR safe (Life Technologies, CA, USA) and visualised using a Gel Doc Software system (BioRad, Hercules, CA, USA).

**Table 5:** Sample metadata of BMSB collection. N represents the number of individuals

Country	Locality	N
Austria	Vienna City	16
Chile	Santiago City	19
China	Anhui Province	6
	Beijing City	26
	Guizhou Province	1
	Hainan Province	7
	Hebei Province	8
	Jilin Province	3
	Shaanxi Province	26
	Shanxi Province	7
Georgia	Eki City	30
	Samegrelo-Zemo Svaneti Region	3
Hungary	Budapest City	38
	Debrecen City	10
	Pécs City	6
	Szeged City	3
	Szombathely City	12
Italy	Codroipo (UD) Comune	3
	Mantua City	1
	Pozzuolo Del Friuli (UD) Comune	11
	Trentino-Alto Region	1
Japan	Akita Prefecture	3
	Chiba Prefecture	2
	Gifu City	2
	Ibaraki Prefecture	2
	Ishikawa Prefecture	2
	Iwate Prefecture	5
	Kagoshima City	8
	Kanagawa Prefecture	3
	Kyoto City	10
	Mie Prefecture	5
	Miyagi Prefecture	3
	Saga Prefecture	3
	Shizuoka City	7
	Yamanashi Prefecture	12
Romania	Bucharest City	22
Serbia	Senta Town	10
Slovenia	Ljubljana City	16
Turkey	Arhavi Town	12
the USA	Maryland State	16
	West Virginia State	9
Total numbers		389

### Selection of restriction enzymes in silico for RAD sequencing

The selection of restriction enzyme (RE) is the crucial step for experimental ddRADseq analysis and discovery of the SNPs across the genome for genetic diversity study, thus initial in silico tests on the RE used for the library was conducted. To select the best RE, a simulation based on the BMSB reference genome (submitted to NCBI GenBank as Hhal_2.0 by the i5k Initiative on 15 December 2017) and 15 combinations of nine enzymes (AvaII, EcoRI, MSeI, MspI, NlaIII, PstI, SbfI, SphI, MluCI). A Python script (Additional file 6) was developed to calculate the number of the potential sheared segments that could be generated by in silico digestion of the reference genome using different combination of restriction enzymes.

### Selection of restriction enzymes in vitro: RAD sequencing and bioinformatics analysis

Nine pairs of enzymes which generated higher numbers of segments in in silico analysis or had been used in other published studies [31] were selected. To further confirm the suitability of the RE pairs, two DNA samples were used for ddRADseq and 18 ddRADSeq libraries (nine pairs of enzymes for each sample) were prepared using Illumina® TruSeq DNA Nano following the manufacturer’s instructions and ToBo lab ezRAD Protocol-v3.2 [52]. Before sequencing, all ddRADSeq libraries were assessed using a DNF-474 High Sensitivity NGS fragment kit (Advanced Analytical Technologies, Inc.) on a Fragment Analyzer™ Automated CE System (Agilent, California, USA) for quality control. The sequencing was conducted by Annoroad Gene Technology Co., Ltd. (Beijing, China) on a HiSeq X Ten sequencing platform (paired end, 2 × 150 bp). The 3’end adaptors of raw reads were trimmed using AdaptorRemoval v2 [53] and reads with phread quality score of less than 20 and length of less than 50 bp were discarded using an in-house python script (Additional file 6). Then, quality-trimmed sequence datasets, were mapped to the BMSB reference genome, Hhal_2.0, using Borows-Wheeler Aligner (BWA) version 0.7.17 [54] in default setting and files with the mapping information (i.e. in SAM format) were converted to BAM format, sorted and indexed using SAMtools version 1.9 [55] before identifying the SNPs. SNPs were called using GATK v4.1 [56]. A stringent SNP filtering was conducted to discard the false positive SNPs as recommended by GATK developers [(QD (QualByDepth) < 2.0, FS (FisherStrand) > 200.0, SOR (StrandOddsRatio) > 10.0, MQRankSum (MappingQualityRankSumTest) < − 12.5, ReadPosRankSum (ReadPosRankSumTest) < − 8.0)]. Population level quality control of SNPs was conduncted using Plink 1.9 (parameters used: call rate 0.8) [57] for subsequent analysis. All bioinformatics analysis was performed on the high-performance computing platform of the New Zealand eScience Infrastructure (NeSI), Auckland, New Zealand.

### Library preparation and ddRAD sequencing

After selecting the most suitable pairs of restriction enzymes (EcoRI and MspI) for digestion of BMSB genome via ddRADseq approach, 399 BMSB individuals were further subjected to double-digest restriction-associated DNA (ddRAD) sequencing. ddRAD libraries were prepared using 500 ng of genomic DNA from each sample following the protocol described by Peterson et al. [31] with some modifications. Briefly, genomic DNA was digested at 37 °C for 5 h using 10 Units of the two selected restriction enzymes, namely EcoRI and MspI (NEB, MA, USA), and deactivated at 65 °C for 20 mins, then followed by the ligation of Illumina adapter sequences and unique 8 bp barcodes, which varied by at least three bases. Sets of 24 differentially barcoded individuals were pooled and run on a 1% agarose gel, where 220 ~ 450 bp fragments were manually excised and purified using a Zymoclean™ Gel DNA recovery kit (Zymo Research, CA, USA). Each pool was amplified by PCR reactions, which were carried out at a total volume of 25 μl, each containing 5 μl of 5 × Reaction buffer, 5 μl of 5 × High GC enhancer, 0.25 μl of Q5 polymerase, 5 nM of library DNA and a unique indexing primer for each pool that corresponds to the standard Illumina multiplexed sequencing protocol. Temperature cycling for PCR comprised one initial denaturation at 98 °C for 30 s, followed by 14 cycles of denaturation at 98 °C for 15 s, annealing at 65 °C for 30 s, and extension at 72 °C for 30 s, followed by a final extension at 72 °C for 5 mins. The PCRs were carried out in a Veriti 96-well thermal cycler (Applied Biosystems, California, USA). DNA libraries were quantified using the Agilent high-sensitivity DNA kit in a 2100 Bioanalyser (Agilent Technologies, CA, USA). Library pools were combined in an equimolar concentration to form a single genomic library and sequenced in one lane of a HiSeq X Ten Illumina sequencer (paired-end, 2 × 150 bp) by Personalbio co. (Shanghai, China).

### Sequencing data pre-processing and quality control of ddRAD data

To pre-process the raw sequencing data generated via ddRAD, a similar pipeline and data quality filtering approach was applied as described in the section of "Selection of restriction enzymes *in vitro*: RAD sequencing and bioinformatics analysis". To call SNPs from ddRADseq data, the raw fastq files were mapped to the scaffold of the reference genome, Hhal_2.0 (RefSeq assembly accession: GCF_000696795.2), to produce a mapping file (SAM format) using BWA v 0.7.17 [54]. The SAM mapping file was converted into binary format (BAM), sorted and indexed to increase calculation speed for further analysis using Samtools v1.9 [55]. SNPs were called from BAM files using GATK4 [56]. The quality control parameter used here was the same as that used for the selection of restriction enzymes above. In addition, the obtained SNPs were further filtered using SNP call rate of 1 and MAF (minor allele frequency) of 0.03 using Plink 1.9 [57]. As a result, 10 samples were discarded due to lower SNP call rate.

### Population genomic analysis

Using the SNPs identified from the 389 BMSB individuals, we conducted principal component analysis (PCA), neighbour-net tree and population structure analysis using fastSTRUCTURE to elucidate the population structure and genetic diversity among the BMSB populations. PCA was conducted based on 1775 high quality SNPs obtained from the 389 individuals using Plink 1.9 [57] (parameters used: --allow-extra-chr --pca) and plotted using R package ggplot [32]. The k-mean was 2 as elbow point. To further explore the genetic relatedness among the BMSB individuals, a Minimum Spanning Network (MSN) based on the SNP results of each individual was also reconstructed using R package poppr [33].

To test for the presence of population structure, pairwise F_ST_ values were generated from the ddRAD sequence data by combining the individual data from the same country as one geographic group. The analyses were implemented in Arlequin 3.5 [58] by converting the SNP profile (in Plink format) to Arlequin format using PGDSpider 2.1.1.5 [59]. The Fst and *p* values were obtained using 110 permutations in Arlequin 3.5. To visualize the genetic relationships, the obtained population average pairwise F_ST_ values were further used to construct a neighbour-net method tree using SplitsTree 4 [34].

The AMOVA calculated the variance components and their statistical significance levels for variations among and within the 12 geographic countries and the five genetic clusters. The AMOVA was conducted by using the distance matrix under 1000 premutation in Arlequin 3.5. To further test the genetic variation, the heterozygosity analysis was preformed using GenAlEx 6.5 [60, 61]. The genotype information of all sample was stored into a excel with GenAlEx 6.5 required format [60, 61]. All calculations were conducted based on the default setting.

To provide additional insight into the genetic variation and population differences, population genetic structure analysis was conducted. The population genetic structure was inferred using the SNPs profile of individual samples. In this process, fastSTRUCTURE 1.0 [62] was used to conduct model-based clustering of all individuals. The fastSTRUCTURE 1.0 was run with the default convergence criterion of 10^− 6^, a simple prior, and ten replicate runs per dataset. The best K value (i.e. the number of populations or clusters that the samples are best divided into) was determined as 5 using a python script, chooseK.py within the fastSTRUCTURE software.

A roadmap of the BMSB invasive pathway was created using Tableau [63] by inputting a excel file with known BMSB background information. Then, the dotted lines and arrow were added in the map using Mac Preview 11.0.

## Supplementary Information

**Additional file 1.** Sequence result of selection of restriction enzymes.

**Additional file 2.** SNP distribution in Scaffold.

**Additional file 3.** PCA plot using one SNP from each scaffold.

**Additional file 4.** 3D PCA plot.

**Additional file 5.** Sample collection information.

**Additional file 6.** RE digest simulation script.

**Additional file 7.** Heterozygosity analysis.

## Data Availability

The raw sequences data were deposited to NCBI Sequence Read Archive (SRA) database under the project ID: PRJNA675311. The reference genome (GCF_000696795.2) was downloaded from NCBI.

## Abbreviations

BMSB: Brown marmorated stink bug
mtDNA: Mitochondrial DNA
COI: Cytochrome c oxidase I
COII: Cytochrome c oxidase II
RADseq: Restriction-site associated DNA sequencing
ddRAD: Double-digest Restriction-Associated DNA
SNPs: Single Nucleotide Polymorphisms
PCA: Principal Component Analysis
MSN: Minimum Spanning Networks
MPI: Ministry for Primary Industries

## References

[1] MacLellan R (2013). Plants and environment brown marmorated stink bug: a potential risk to New Zealand. Surveillance..

[2] Hoebeke ER, Carter ME (2003). *Halyomorpha halys* (Stǻl) (Heteroptera: Pentatomidae): a polyphagous plant pest from Asia newly detected in North America. Proc Entomol Soc Wash.

[3] Nielsen AL, Hamilton GC (2009). Life history of the invasive species *Halyomorpha halys* (Hemiptera: Pentatomidae) in northeastern United States. Ann Entomol Soc Am.

[4] Leskey TC, Nielsen AL (2018). Impact of the invasive brown marmorated stink bug in North America and Europe: history, biology, ecology, and management. Annu Rev Entomol.

[5] Josifov M, IM K. (1978). heteroptera aus korea. Ii. Aradidae, berytidae, lygaeidae, pyrrhocoridae, rhopalidae, alydidae, coreidae, urostylidae, acanthosomatidae, sautelleridae, pentatomidae, cydnidae, plataspidae. Fragm. Faun..

[6] Rider DA, Zheng LY, Kerzhner IM (2002). Checklist and nomenclatural notes on the Chinese Pentatomidae (Heteroptera). II Pentatominae Zoosyst Ross.

[7] Lee D, Short BD, Joseph SV, Bergh JC, Leskey TC (2013). Review of the biology, ecology, and management of *Halyomorpha halys* (Hemiptera: Pentatomidae) in China, Japan, and the Republic of Korea. Environ Entomol.

[8] EPPO (2020). *Halyomorpha halys* (HALYHA). EPPO Global database.

[9] Zhu G, Bu W, Gao Y, Liu G (2012). Potential geographic distribution of brown marmorated stink bug invasion (*Halyomorpha halys*). PLoS One.

[10] Macavei LI, Baetan R, Oltean I, Florian T, Varga M, Costi E (2015). First detection of *Halyomorpha halys* Stål, a new invasive species with a high potential of damage on agricultural crops in Romania. Lucrări Ştiinţifice, Universitatea de Stiinte Agricole Şi Medicină Veterinară “Ion Ionescu de la Brad” Iaşi. Seria Agronomie.

[11] Heckmann R (2012). First evidence of *Halyomorpha halys* (Stal, 1855) (Heteroptera: Pentatomidae) in Germany. Heteropteron..

[12] Vetek G, Papp V, Haltrich A, Redei D (2014). First record of the brown marmorated stink bug, *Halyomorpha halys* (Hemiptera: Heteroptera: Pentatomidae), in Hungary, with description of the genitalia of both sexes. Zootaxa..

[13] Maistrello L, Dioli P, Vaccari G, Nannini R, Bortolotti P, Caruso S, et al. First records in Italy of the Asian stinkbug *Halyomorpha halys*, a new threat for fruit crops. Atti, Giornate Fitopatologiche, Chianciano Terme (Siena), 18–21 marzo 2014, Volume primo. 2014; 283–288.

[14] Wermelinger B, Wyniger D, Forster (2008). First records of an invasive bug in Europe: *Halyomorpha halys* Stal (Heteroptera: Pentatomidae), a new pest on woody ornamentals and fruit trees?. Mitt Schweiz Entomol Ges.

[15] Milonas PG, Partsinevelos GK (2014). First report of brown marmorated stink bug *Halyomorpha halys* Stål (Hemiptera: Pentatomidae) in Greece. EPPO Bull.

[16] Cesari M, Maistrello L, Ganzerli F, Dioli P, Rebecchi L, Guidetti R (2015). A pest alien invasion in progress: potential pathways of origin of the brown marmorated stink bug *Halyomorpha halys* populations in Italy. J Pest Sci.

[17] Faúndez EI, Rider DA (2017). The brown marmorated stink bug *Halyomorpha halys* (Stål, 1855) (Heteroptera: Pentatomidae) in Chile. Arquivos Entomolóxicos.

[18] Kriticos DJ, Kean JM, Phillips CB, Senay SD, Acosta H, Haye T (2017). The potential global distribution of the brown marmorated stink bug, *Halyomorpha halys*, a critical threat to plant biosecurity. J Pest Sci.

[19] Gariepy TD, Haye T, Fraser H, Zhang J (2014). Occurrence, genetic diversity, and potential pathways of entry of *Halyomorpha halys* in newly invaded areas of Canada and Switzerland. J Pest Sci.

[20] Labware LIMS (2020). Ministry for primary industries.

[21] Vandervoet TF, Bellamy DE, Anderson D, MacLellan R (2019). Trapping for early detection of the brown marmorated stink bug, *Halyomorpha halys*, in New Zealand. N Z Plant Prot.

[22] Xu J, Fonseca DM, Hamilton GC, Hoelmer KA, Nielsen AL (2014). Tracing the origin of US brown marmorated stink bugs, *Halyomorpha halys*. Biol Invasions.

[23] Zhu G, Ye Z, Du J, Zhang D, Zhen Y, Zheng C (2016). Range wide molecular data and niche modeling revealed the Pleistocene history of a global invader (*Halyomorpha halys*). Sci Rep.

[24] Morrison WR, Milonas P, Kapantaidaki DE, Cesari M, Di Bella E, Guidetti R (2017). Attraction of *Halyomorpha halys* (Hemiptera: Pentatomidae) haplotypes in North America and Europe to baited traps. Sci Rep.

[25] Hebert PD, Cywinska A, Ball SL, Dewaard JR (2003). Biological identifications through DNA barcodes. Proc R Soc B.

[26] Kang J, Ma X, He S (2017). Population genetics analysis of the Nujiang catfish Creteuchiloglanis macropterus through a genome-wide single nucleotide polymorphisms resource generated by RAD-seq. Sci Rep.

[27] Davey JW, Hohenlohe PA, Etter PD, Boone JQ, Catchen JM, Blaxter ML (2011). Genome-wide genetic marker discovery and genotyping using next-generation sequencing. Nat Rev Genet.

[28] Lemopoulos A, Prokkola JM, Uusi-Heikkilä S, Vasemägi A, Huusko A, Hyvärinen P, Koljonen ML, Koskiniemi J, Vainikka A (2019). Comparing RADseq and microsatellites for estimating genetic diversity and relatedness - implications for brown trout conservation. Ecol Evol.

[29] Miller MR, Dunham JP, Amores A, Cresko WA, Johnson EA (2007). Rapid and cost-effective polymorphism identification and genotyping using restriction site associated DNA (RAD) markers. Genome Res.

[30] Lexer C, Wüest RO, Mangili S, Heuertz M, Stölting KN, Pearman PB, Forest F, Salamin N, Zimmermann NE, Bossolini E (2014). Genomics of the divergence continuum in an African plant biodiversity hotspot, I: drivers of population divergence in Restio capensis (Restionaceae). Mol Ecol.

[31] Peterson BK, Weber JN, Kay EH, Fisher HS, Hoekstra HE (2012). Double digest RADseq: an inexpensive method for de novo SNP discovery and genotyping in model and non-model species. PLoS One.

[32] Wickham H (2016). ggplot2: elegant graphics for data analysis. Springer-Verlag New York. ISBN 978-3-319-24277-4.

[33] Kamvar ZN, Tabima JF, Grünwald NJ. Poppr: an R package for genetic analysis of populations with clonal, partially clonal, and/or sexual reproduction. PeerJ. 2014;2:e281. 10.7717/peerj.281. 10.7717/peerj.281 PMC3961149 24688859

[34] Huson DH, Bryant D. Application of phylogenetic networks in evolutionary studies. Mol Biol Evol. 2006;23(2):254–67. 10.1093/molbev/msj030. 10.1093/molbev/msj030 16221896

[35] Imamura K. Prehistoric Japan: new perspectives on insular East Asia: University of Hawaii Press; 1996.

[36] Wiman NG, Walton VM, Shearer PW, Rondon SI, Lee JC (2015). Factors affecting flight capacity of brown marmorated stink bug, *Halyomorpha halys* (Hemiptera: Pentatomidae). J Pest Sci.

[37] Valentin RE, Nielsen AL, Wiman NG, Lee D, Fonseca DM (2017). Global invasion network of the brown marmorated stink bug, *Halyomorpha halys*. Sci Rep.

[38] Yan J, Pal C, Anderson D, Vetek G, Farkas P, Burne A (2020). Genetic diversity analysis of brown marmorated stink bug, *Halyomorpha halys* based on mitochondrial COI and COII haplotypes. BMC Genet.

[39] Fu Y (1997). Statistical tests of neutrality of mutations against population growth, hitchhiking and background selection. Genetics..

[40] Cesari M, Maistrello L, Piemontese L, Bonini R, Dioli P, Lee W, Park CG, Partsinevelos GK, Rebecchi L, Guidetti R (2018). Genetic diversity of the brown marmorated stink bug *Halyomorpha halys* in the invaded territories of Europe and its patterns of diffusion in Italy. Biol Invasions.

[41] Leskey TC, Hamilton GC, Nielsen AL, Polk DF, Rodriguez-Saona C, Bergh JC, Herbert DA, Kuhar TP, Pfeiffer D, Dively GP, Hooks CRR, Raupp MJ, Shrewsbury PM, Krawczyk G, Shearer PW, Whalen J, Koplinka-Loehr C, Myers E, Inkley D, Hoelmer KA, Lee DH, Wright SE (2012). Pest status of the brown marmorated stink bug, *Halyomorpha halys* in the USA. Outlooks Pest Manag.

[42] Haye T, Wyniger D, Gariepy T, Müller G, Pospischil R, Robinson WH (2014). Recent range expansion of brown marmorated stink bug in Europe. Proceedings of the 8^th^ International Conference on Urban Pests. Zurich, Switzerland, July 20–23, 2014.

[43] Cianferoni F, Graziani F, Dioli P, Ceccolini F (2018). Review of the occurrence of *Halyomorpha halys* (Hemiptera: Heteroptera: Pentatomidae) in Italy, with an update of its European and world distribution. Biologia..

[44] Gapon DA (2016). First records of the brown marmorated stink bug *Halyomorpha halys* (Stål, 1855) (Heteroptera, Pentatomidae) in Russia, Abkhazia, and Georgia. Entomol Rev.

[45] Claerebout S, Haye T, Ólafsson E, Pannier E, Bultot J (2018). Première occurrence de *Halyomorpha halys* (Stål, 1855) (Hemiptera: Heteroptera: Pentatomidae) pour la Belgique et actualisation de sa distribution en Europe. Bull Soc R Belg Entomol.

[46] Ciceoi R, Bolocan IG, Dobrin I (2017). The spread of brown marmorated stink bug, *Halyomorpha halys*, in Romania. J Hortic Sci Biotechnol.

[47] Rabitsch W, Friebe GJ (2015). From the west and from the east? First records of *Halyomorpha halys* (Stål, 1855) (Hemiptera: Heteroptera: Pentatomidae) in Vorarlberg and Vienna, Austria. Beiträge zur Entomofaunistik..

[48] Šeat J (2015). *Halyomorpha halys* (Stål, 1855) (Heteroptera: Pentatomidae) a new invasive species in Serbia. Acta Entomol Serbica.

[49] Rot M, Devetak M, Carlevaris B, Žežlina J, Žežlina I (2018). First record of brown marmorated stink bug (*Halyomorpha halys* Stål, 1855) (Hemiptera: Pentatomidae) in Slovenia. Acta Entomol Sloven.

[50] Güncan A, Gümüş E (2019). Brown marmorated stink bug, *Halyomorpha halys* (StåL, 1855) (Hemiptera: Heteroptera, Pentatomidae), a new and important pest in Turkey. Entomol News.

[51] Sparks ME, Bansal R, Benoit JB, Blackburn MB, Chao H, Chen M, Cheng S, Childers C, Dinh H, Doddapaneni HV, Dugan S, Elpidina EN, Farrow DW, Friedrich M, Gibbs RA, Hall B, Han Y, Hardy RW, Holmes CJ, Hughes DST, Ioannidis P, Cheatle Jarvela AM, Johnston JS, Jones JW, Kronmiller BA, Kung F, Lee SL, Martynov AG, Masterson P, Maumus F, Munoz-Torres M, Murali SC, Murphy TD, Muzny DM, Nelson DR, Oppert B, Panfilio KA, Paula DP, Pick L, Poelchau MF, Qu J, Reding K, Rhoades JH, Rhodes A, Richards S, Richter R, Robertson HM, Rosendale AJ, Tu ZJ, Velamuri AS, Waterhouse RM, Weirauch MT, Wells JT, Werren JH, Worley KC, Zdobnov EM, Gundersen-Rindal DE (2020). Brown marmorated stink bug, *Halyomorpha halys* (Stål), genome: putative underpinnings of polyphagy, insecticide resistance potential and biology of a top worldwide pest. BMC Genomics.

[52] Toonen RJ, Puritz JB, Forsman ZH, Whitney JL, Fernandez-Silva I, Andrews KR (2013). ezRAD: a simplified method for genomic genotyping in non-model organisms. PeerJ.

[53] Schubert M, Lindgreen S, Orlando L (2016). AdapterRemoval v2: rapid adapter trimming, identification, and read merging. BMC Res Notes.

[54] Li H, Durbin R (2009). Fast and accurate short read alignment with burrows–wheeler transform. Bioinformatics..

[55] Li H, Handsaker B, Wysoker A, Fennell T, Ruan J, Homer N, Marth G, Abecasis G, Durbin R, 1000 Genome Project Data Processing Subgroup (2009). The sequence alignment/map format and SAMtools. Bioinformatics..

[56] McKenna A, Hanna M, Banks E, Sivachenko A, Cibulskis K, Kernytsky A, et al. The genome analysis toolkit: a MapReduce framework for analyzing next-generation DNA sequencing data. Genome Res. 2010;20(9):1297–303. 10.1101/gr.107524.110. 10.1101/gr.107524.110 PMC2928508 20644199

[57] Chang CC, Chow CC, Tellier LC, Vattikuti S, Purcell SM, Lee JJ. Second-generation PLINK: rising to the challenge of larger and richer datasets. Gigascience. 2015;4(1):7. 10.1186/s13742-015-0047-8. 10.1186/s13742-015-0047-8 PMC4342193 25722852

[58] Excoffier L, Lischer HE. Arlequin suite ver 3.5: a new series of programs to perform population genetics analyses under Linux and windows. Mol Ecol Resour. 2010;10(3):564–7. 10.1111/j.1755-0998.2010.02847.x. 10.1111/j.1755-0998.2010.02847.x 21565059

[59] Lischer HE, Excoffier L. PGDSpider: an automated data conversion tool for connecting population genetics and genomics programs. Bioinformatics. 2011;28(2):298–9. 10.1093/bioinformatics/btr642. 10.1093/bioinformatics/btr642 22110245

[60] Peakall R, Smouse PE (2006). GENALEX 6: genetic analysis in excel. Population genetic software for teaching and research. Mol Ecol Notes.

[61] Peakall R, Smouse PE (2012). GenAlEx 6.5: genetic analysis in excel. Population genetic software for teaching and research-an update. Bioinformatics.

[62] Raj A, Stephens M, Pritchard JK (2014). fastSTRUCTURE: variational inference of population structure in large SNP data sets. Genetics..

[63] Deardorff A (2016). Tableau (version. 9.1). JMLA..

