# Supplementary figures and images for: ddRAD sequencing: an emerging technology added to the biosecurity toolbox for tracing the origin of brown marmorated stink bug, Halyomorpha halys (Hemiptera: Pentatomidae)
Source: BMC Genomics. 2021 May 17;22:355. doi: 10.1186/s12864-021-07678-z (PMC8130256; doi:10.1186/s12864-021-07678-z)

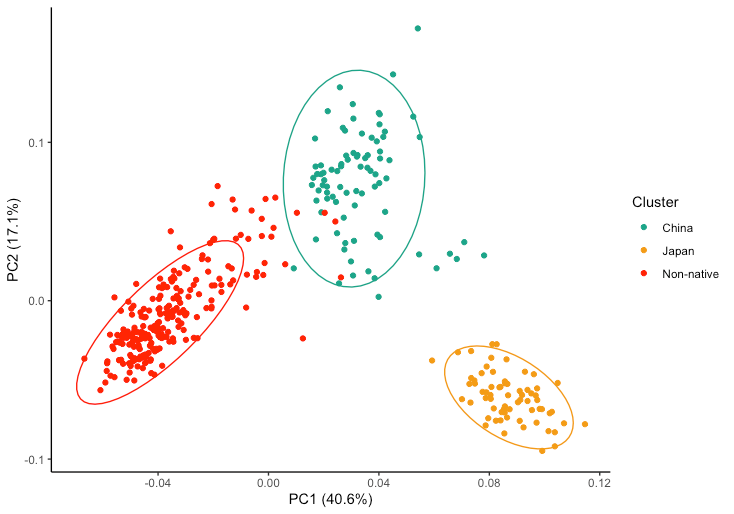

Supplement: Supplementary file 3 — Additional file 3. PCA plot using one SNP from each scaffold. [file 12864_2021_7678_MOESM3_ESM.png]
